# Identification of miR171a-*GRAS50* Regulatory Module Associated with Wood Properties in *Populus tomentosa*

**DOI:** 10.3390/ijms27010228

**Published:** 2025-12-25

**Authors:** Guhang Shi, Rui Huang, Shitong Qin, Mingyang Quan, Deqiang Zhang

**Affiliations:** 1 State Key Laboratory of Tree Genetics and Breeding, College of Biological Sciences and Technology, Beijing Forestry University, Beijing 100083, China; a84608677@163.com (G.S.); huangrui19901@163.com (R.H.); qinshitong1998@163.com (S.Q.); mingyangquan@bjfu.edu.cn (M.Q.); 2 National Engineering Research Center of Tree Breeding and Ecological Restoration, College of Biological Sciences and Technology, Beijing Forestry University, Beijing 100083, China

**Keywords:** *Populus tomentosa*, miR171, GRAS, association analysis, DAP-seq

## Abstract

Enhancing wood properties, particularly fiber length (FL), represents a critical objective in *Populus tomentosa* breeding programs. However, the molecular mechanisms regulating these traits remain largely elusive. Here, an integrative analysis of the PtomiR171 family, uncovering substantial functional divergence among PtomiR171 family members and identified a *PtomiR171a-PtoGRAS50* regulatory axis that may control cellulose-related gene expression and influence fiber development in *P. tomentosa*. Single-nucleotide polymorphism (SNP)-based association studies implicated the role of the *PtomiR171a-PtoGRAS50* module in modulating FL. Combined with dual-luciferase reporter gene assay, real-time reverse transcription polymerase chain reaction (RT-qPCR), transcriptome and degradome analysis, *PtomiR171a* exerts a negative regulatory effect on *PtoGRAS50*, which is a key regulator of early xylem development. DNA affinity purification sequencing (DAP-seq) identified two downstream putative target genes of *PtoGRAS50*, both of which are involved in cellulose biosynthesis and metabolism. Unlike previous studies about miRNAs in *P. tomentosa*, this work narrows its scope to miR171 and elucidates the downstream regulatory module. Collectively, these findings elucidate a critical *PtomiR171a-PtoGRAS50* regulatory axis, advancing our understanding of the genetic networks that orchestrate wood properties, deepening insights into FL modulation, and laying a foundation for the development of targeted genetic strategies to enhance wood quality in *P. tomentosa*.

## 1. Introduction

Wood constitutes the primary reservoir of terrestrial biomass production and a renewable feedstock for the timber, paper, and bioenergy industries. Fast-growing poplars are among the most extensively cultivated sources of plantation wood, and *Populus tomentosa* is an economically important species in temperate forestry. Wood formation is a complex, continuous developmental program governed by hierarchical transcriptional networks; within these networks, microRNAs (miRNAs) fine-tune transcription factor activity to regulate cell wall biosynthesis and secondary growth. High-throughput small-RNA sequencing and resequencing studies have identified multiple miRNAs that influence fibre development across angiosperms—for example, *miR828*/*miR858* modulate MYB-mediated processes in *Arabidopsis*, and *miR396b* knockdown alters fibre length in cotton [1,2]. However, the contribution of miR171 to wood quality traits remains poorly characterized in *P. tomentosa*, motivating focused investigation in this species.

The miR171 family is highly conserved across plants and predominantly targets members of the GAI-RGA-and-SCR (GRAS) transcription factor family, including regulators such as hairy meristem (HAM) [3], nodulation signaling pathway 2 (NSP2), and scarecrow-like (SCL). The miR171 cooperates with the WUSCHEL (WUS) and CLAVATA3 (CLV3) feedback loop to maintain stem cell homeostasis by regulating *HAM1* and *HAM2* expression [4,5]. In *Medicago truncatula*, *miR171h* specifically targets *NSP2* transcripts, thereby playing a key role in regulating cambial mycorrhizal root colonization and the nodulation signaling pathway [6]. Despite these insights, the specific genes targeted by miR171 in perennial woody plants and the consequent effects on wood formation remain largely unexplored, representing a significant avenue for developing novel strategies to enhance wood properties.

Deciphering the genetic architecture of wood formation is a prerequisite for rational improvement of biomass quantity and quality. Advances in genome sequencing, dense genotyping and the availability of high-quality *Populus* reference genomes now permit population-scale dissection of the quantitative loci underlying wood traits [7]. Candidate gene association analyses provide a powerful, hypothesis-free strategy to map loci associated with complex traits and have been successfully applied across crops and model species to reveal loci and regulatory elements controlling diverse phenotypes [8]. Although association analyses are powerful for discovering loci linked to complex traits, statistical associations alone do not establish molecular mechanisms or causality. Therefore, candidate-module association analysis with targeted functional assays needs to be combined to dissect the role of a specific miRNA-target module in wood properties. By integrating a comprehensive strategy, researchers can enhance phenotypic prediction accuracy and construct a more holistic genetic framework for traits such as wood formation [9].

Our previous small RNA sequencing (sRNA-seq) analysis identified miR171 as a key player in the formation of specialized wood in *P. tomentosa* [10]. Building on this foundation, the present study provides a comprehensive examination of the PtomiR171 family, with a focus on their distribution patterns, sequence features, and expression profiles. To elucidate their regulatory roles, we employed degradome sequencing to identify target genes of PtomiR171. To further investigate the impact of miR171 and its target genes across different stages of wood development, we performed RNA sequencing (RNA-seq) on xylem tissues collected at various developmental stages. Through candidate gene association analysis, based on allelic variation within the PtomiR171-target module, we established the interaction between *PtomiR171a* and *PtoGRAS50* as a key regulatory mechanism influencing wood properties. This relationship was further substantiated using dual-luciferase reporter assays, which confirmed a direct regulatory link. Moreover, we employed DNA affinity purification sequencing (DAP-seq) and identified two downstream targets of *PtoGRAS50*, both implicated in cellulose biosynthesis and metabolism, providing further insights into the molecular pathways shaping wood development. Collectively, these findings shed light on the intricate potential regulatory network mediated by the *PtomiR171a-PtoGRAS50*, offering a deeper understanding of the genetic mechanisms underpinning wood development in *P. tomentosa* and laying the groundwork for future advancements in improving economically significant wood traits.

## 2. Results

### 2.1. Genome-Wide Identification and Expression Analysis of miR171 in Populus Tomentosa

**Figure 1:**
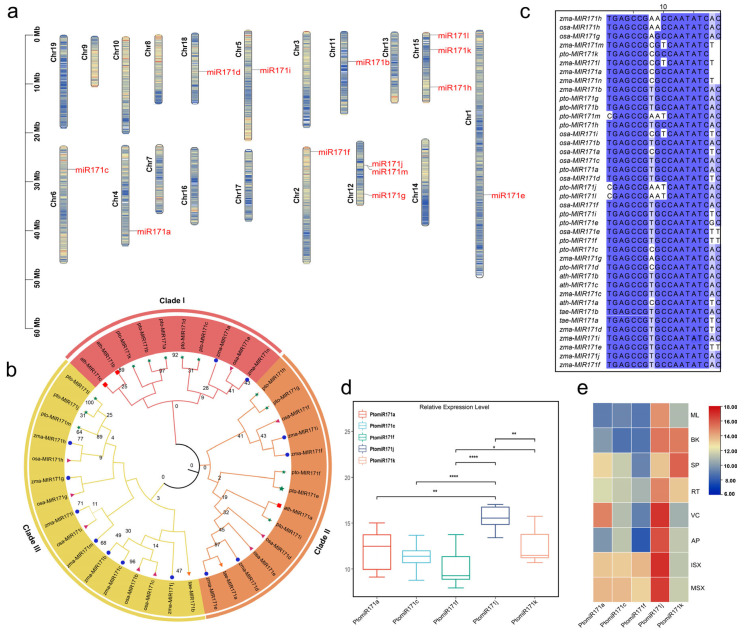
Genome-wide identification and expression analysis of miR171 in *Populus tomentosa.* (**a**) Chromosomal distribution of miR171 family members in *P. tomentosa*. Chromosome coordinates are shown in Mb. Color bar indicates gene density (genes per Mb); (**b**) Phylogenetic analysis of miR171 precursor sequences from five species (*Oryza sativa*, *Arabidopsis thaliana*, *Triticum aestivum*, *P. tomentosa*, and *Zea mays*), constructed using the neighbor-joining method. The major branches are shown in arcs and labeled as Clade I (red), Clade II (orange), and Clade III (yellow). miR171 family members from different species are denoted by distinct shapes and colors, while PtomiR171 members are highlighted with green asterisks; (**c**) Multiple sequence alignment of mature miR171 sequences from these five species, showing sequence conservation among family members. Conserved nucleotides across all sequences are shaded in dark blue, indicating high sequence conservation; (**d**) Expression profiles of miR171 family members displayed as boxplots. *p*-values were calculated using two-sided pairwise comparisons, * *p* < 0.05; ** *p* < 0.01; **** *p* < 0.0001; (**e**) Tissue-specific expression patterns of PtomiR171 family members across eight tissues, visualized in a heatmap. The analysis was performed using poly(A) tailing-based approaches and a common oligo(dT)-based reverse transcription strategy, ensuring comprehensive detection of miR171 expression in *P. tomentosa*.

To characterize the genomic distribution and expression patterns of miR171 precursor sequences in *P. tomentosa*, we identified 13 PtomiR171 precursor sequences based on homology with their counterparts in *Populus trichocarpa*. These sequences were found to be unevenly distributed across nine chromosomes (Table S1), with notable clustering on chromosomes 12 and 15, each harboring three miR171 sequences (Figure 1a). Most PtomiR171 precursor sequences were located in intergenic regions, indicating their likely role as independent transcriptional units. However, *miR171j* and *miR171m* were positioned less than 10 kb apart on the same strand, forming a miRNA cluster. This close genomic proximity suggests that these two miRNAs may be co-transcribed as a single unit, a phenomenon previously observed in other plant species [11]. This clustering could indicate potential functional synergy or coordinated regulation of downstream target genes.

To further explore the evolutionary relationships among miR171 family members across different plant species, we constructed a phylogenetic tree based on the precursor sequences of 40 miR171 family members. This analysis revealed three distinct subgroups: Clade I (ten members), Clade II (14 members), and Clade III (16 members) (Figure 1b). Interestingly, the mature sequences of miR171 displayed a high degree of conservation across species, with two highly conserved motifs, ‘GAGCCG’ and ‘CAATATC’ (Figure 1c), suggesting strong evolutionary constraints on their function. Notably, three *P. tomentosa* members, *PtomiR171j*, *PtomiR171l*, and *PtomiR171m*, clustered within Clade III, which predominantly comprises monocotyledonous miR171 members. This phylogenetic placement indicates that these three PtomiR171 members share a closer evolutionary relationship with miR171 homologs from *Zea mays* and *Oryza sativa* than with their homologs within *P. tomentosa*. Such evolutionary divergence suggests possible functional specialization or retention of ancestral regulatory mechanisms in these specific miR171 members.

To investigate the expression patterns of PtomiR171 precursors, we analyzed the expression levels of five mature miR171 sequences across various tissues. The results revealed significant variation in expression profiles among different miR171 members. Among them, *miR171j* exhibited the highest expression levels across most tissues (Figure 1d), whereas *miR171c* and *miR171f* consistently showed low expression levels, suggesting differential potential regulatory roles within the PtomiR171 family. Specifically, *miR171a* displayed high expression in immature secondary xylem (ISX), mature secondary xylem (MSX), and vascular cambium (VC), while miR171k was predominantly expressed in Secondary phloem (SP) and bark (BK) (Figure 1e). These findings highlight the potential functional diversity of PtomiR171 members across tissues. In particular, the tissue-specific enrichment of *miR171a* and *miR171k* suggests their critical roles in vascular development and wood formation in *P. tomentosa*.

### 2.2. Genome-Wide Identification and Expression Analysis of Target Genes

**Figure 2:**
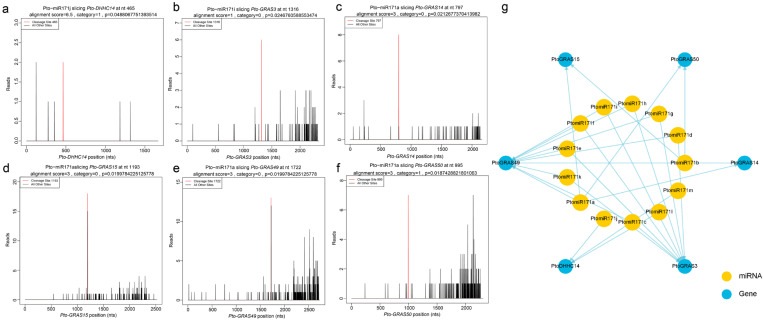
Identification of putative miR171 target genes in *Populus tomentosa* via degradome sequencing: (**a**–**f**) predicted miR171-mediated cleavage sites in six putative target genes: *PtoDHHC14* (**a**), *PtoGRAS3* (**b**), *PtoGRAS14* (**c**), *PtoGRAS15* (**d**), *PtoGRAS49* (**e**), and *PtoGRAS50* (**f**). The X-axis represents the cleavage site positions (nt), while the Y-axis indicates the abundance of raw degradome tags. Red vertical bars highlight the most likely cleavage sites; (**g**) Potential transcript regulatory network of miR171 and its putative target genes. The outer blue circles represent the putative target genes, while the inner yellow circles represent the 13 PtomiR171 family members. Each miRNA-gene pair denotes a predicted regulatory interaction between miRNA and its corresponding target gene.

To uncover the downstream mechanisms of miR171 in *P. tomentosa*, we employed degradome sequencing to identify its putative target genes. The analysis uncovered six primary putative target genes, *PtoGRAS3*, *PtoGRAS14*, *PtoGRAS15*, *PtoGRAS49*, *PtoGRAS50*, and *PtoDHHC14*, encoding GAI-RGA-and-SCR (GRAS) transcription factors (TFs) and a DHHC-type zinc finger protein (Figure 2a–f; Table S2). Our degradome sequencing analysis revealed 27 miRNA-target gene pairs, providing new insights into the miR171 regulatory network in poplar (Figure 2g). Notably, a substantial 88.9% of these pairs represent conserved miRNA-target interactions, consistent with previous findings in other plant species [12]. The analysis further revealed that miR171 exhibits a strong preference for targeting GRAS genes, accounting for 24 out of 27 interactions, while only three pairs involved DHHC genes. Interestingly, these DHHC genes were specifically cleaved by the mature sequence of *PtomiR171j*, suggesting a potential functional divergence within the miR171 family in *P. tomentosa*. This divergence implies that *miR171j* may have acquired a unique potential regulatory role beyond the conventional GRAS-targeting function of miR171, possibly contributing to the specialized aspects of wood development and vascular differentiation.

**Figure 3:**
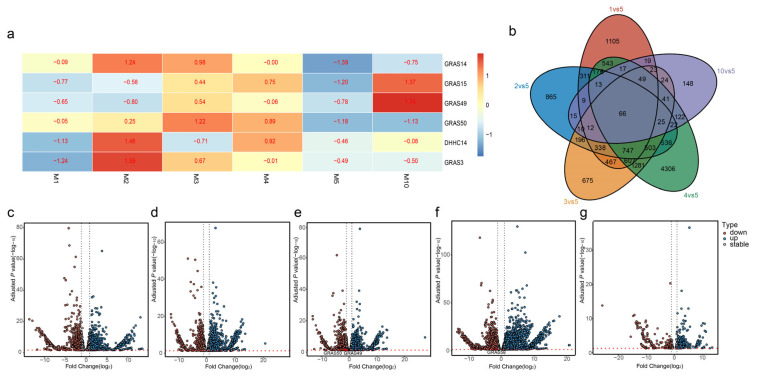
Expression pattern analysis of putative miR171 target genes in *Populus tomentosa*: (**a**) Heatmap showing the expression patterns of putative miR171 target genes across different xylem developmental stages; (**b**) Venn diagrams illustrating the overlap of differentially expressed genes (DEGs) across various comparison groups; (**c**–**g**) Volcano plots displaying DEGs between successive developmental stages: one-year old xylem (m1), two-year old xylem (m2), three-year old xylem (m3), four-year old xylem (m4), five-year old xylem (m5), ten-year old xylem (m10) (**c**) m1 vs. m5; (**d**) m2 vs. m5; (**e**) m3 vs. m5; (**f**) m4 vs. m5; (**g**) m10 vs. m5.

The transition from juvenile wood (JW) to mature wood (MW) is a critical developmental process in *Populus*, typically occurring between 3–5 years for JW and beyond five years for MW [13]. Given that miR171 has previously been implicated in specialized wood formation in *P. tomentosa* [10], we further investigated the transcriptomic dynamics of its putative target genes throughout xylem development in *Populus* at six time points (1, 2, 3, 4, 5, and 10 years old) using three biological replicates. Our analysis revealed distinct temporal expression patterns for putative miR171 target genes (Figure 3a). Specifically, *GRAS15* and *GRAS49* exhibited predominant expression in 10-year-old xylem, suggesting a role in MW formation. In contrast, *DHHC14* and *GRAS3* were highly expressed in 2-year-old xylem, while *GRAS14* showed elevated expression in 2- and 3-year-old xylem, indicating potential functions in early xylem differentiation. *GRAS50*, on the other hand, was mainly expressed in 3- and 4-year-old xylem, aligning with the JW-to-MW transition period. These findings suggest a developmental stage-specific regulation of putative miR171 target genes, emphasizing their distinct roles in xylem tissue formation and wood maturation.

Through pairwise comparative analysis across successive developmental stages in xylem development, we identified a subset of 66 genes that exhibited differential expression across all five comparison groups (Figure 3b). Comparisons between 5-year-old xylem tissues (m5) and those from other developmental stages revealed 4502, 3844, 5064, 9054, and 615 differentially expressed genes (DEGs) (Figure 3c–g), respectively. Notably, the greatest number of DEGs was observed in the comparison between 4-year-old (m4) and m5, suggesting substantial transcriptomic shifts during the juvenile-to-mature wood transition. Conversely, the fewest DEGs were detected in the 10-year-old (m10) vs. m5 comparison, indicating greater transcriptomic stability in mature xylem (Figure 3c–g). Interestingly, *GRAS50* exhibited significant differential expression in both 3-year-old (m3) vs. m5 and m4 vs. m5 comparisons, reinforcing its critical role in the early stages of xylem differentiation (Figure 3c–g). Overall, our findings from degradome sequencing and DEG analysis highlight the role of putative miR171 target genes in orchestrating xylem development and wood formation in *Populus*.

### 2.3. PtomiR171a-PtoGRAS50 Module and Its Association with Fiber Length Traits

**Figure 4:**
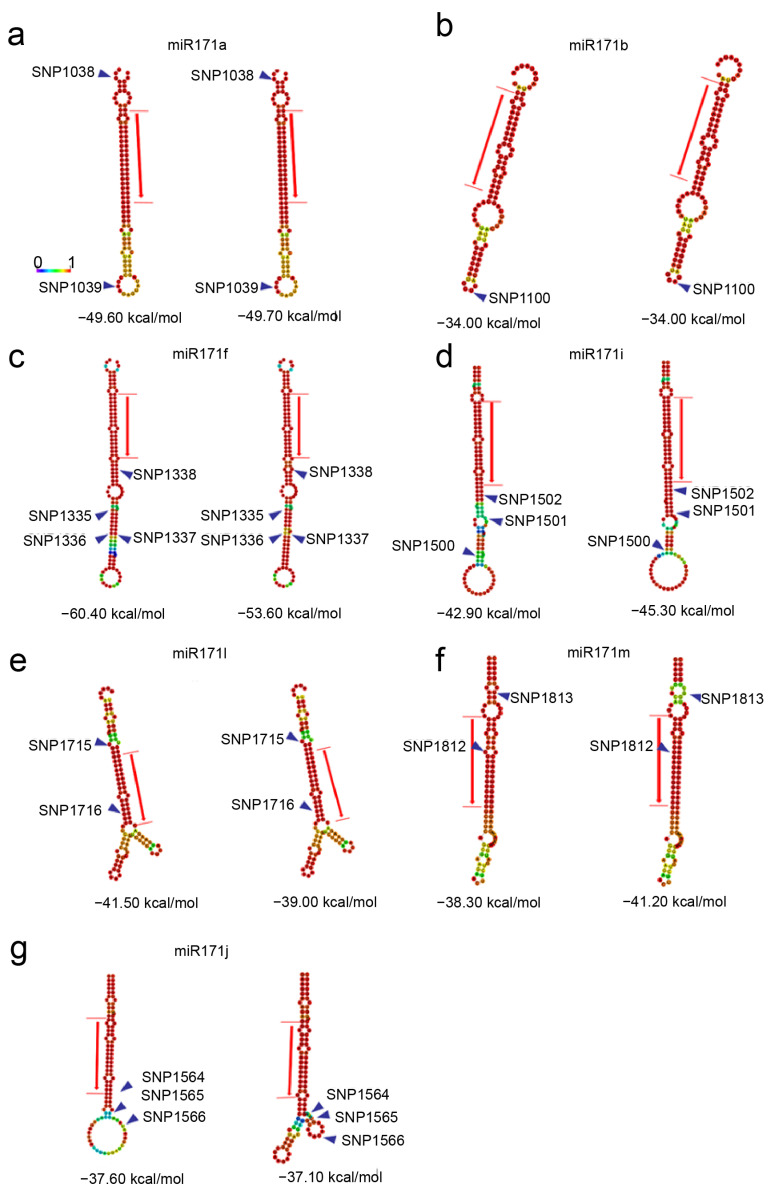
Allelic variants in PtomiR171 precursor sequences: (**a**) Centroid secondary structure of *PtomiR171a*; (**b**) Centroid secondary structure of *PtomiR171b*; (**c**) Centroid secondary structure of *PtomiR171f*; (**d**) Centroid secondary structure of *PtomiR171i*; (**e**) Centroid secondary structure of *PtomiR171l*; (**f**) Centroid secondary structure of *PtomiR171m*; (**g**) Centroid secondary structure of *PtomiR171j*.

To identify single-nucleotide polymorphisms (SNPs) within the 13 PtomiR171 family members, we analyzed 303 genetically unrelated individuals from the association population. A total of 839 common SNPs were identified, with a minor allele frequency of >5% and a miss rate of <20% across 303 accessions. Seven miR171 members contain SNPs. Among these, one miR171 member (*miR171b*) harbored an SNP within its mature sequences, while six others contained multiple SNPs per sequence. To assess the structural impact of SNPs on miRNA171 hairpin conformations, we calculated the energy change (ΔΔG) between reference and altered sequences using the RNAfold Program. Our analysis revealed that SNPs can disrupt or introduce new base pairings, consequently modifying the hairpin conformation and altering free energy stability (Figure 4) in seven pre-miR171s. Specifically, the minimum free energy (MFE) of miR171a, miR171i, and miR171m decreased, indicating enhanced structural stability (Figure 4). Conversely, *PtomiR171j* exhibited a significant shift in its stem-loop structure, altering the predicted secondary conformation (MFE change from −37.60 to −37.10 kcal/mol). These findings suggest that SNP-induced modifications in the secondary structure of pre-miRNAs may influence precursor stability and processing efficiency, ultimately affecting miR171 accumulation and its regulatory function in wood development.

**Figure 5:**
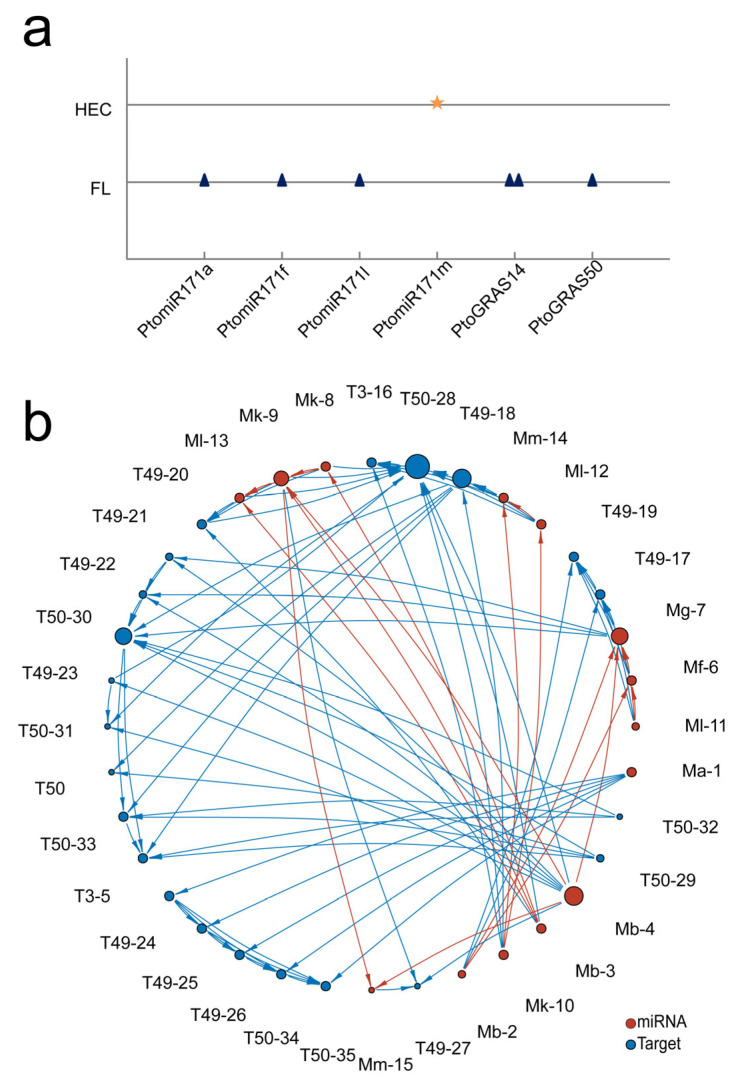
Genetic effects of PtomiR171 members and their putative target genes: (**a**) SNP-trait association analysis of miR171 and its putative target genes. Yellow star and blue triangle represent distinct SNP loci, with the Y-axis representing eight wood quality traits and the X-axis indicating the genomic locations of allelic variants. The shape at the intersection of a gene and a trait indicates a significant SNP-trait association, based on additive and dominant effects; (**b**) Epistasis network analysis of PtomiR171 family members and their putative target genes. Blue circles indicate SNPs in target genes, while red circles indicate intermediate variants in the miR171 family member.

To identify candidate genes associated with wood property traits, we analyzed 1843 SNPs located within PtomiR171 family members and their putative target genes. Our association analysis revealed seven significant SNP-trait associations (*Q* < 0.1), corresponding to seven SNPs and two wood traits (Fiber Length and Hemicellulose content) (Table 1; Figure 5a). Additionally, *PtoGRAS50*_SNP993, situated within the flanking region of *PtoGRAS50*, was associated with the Fiber Length (FL). Furthermore, PtomiR171a_SNP1040 also exhibited a strong association with FL, reinforcing the hypothesis that *PtomiR171a* and its target gene *PtoGRAS50* may co-regulate wood formation.

To further explore potential epistatic interactions among PtomiR171 members and their candidate target genes within the GRAS and DHHC gene families, we conducted epistatic interaction analyses across eight wood property traits. A total of 96 significant pairwise epistatic associations were identified (Figure 5b), including five unique SNPs in *PtomiR171a* and 42 unique SNPs in *PtoGRAS50* (Table S3). The pairwise effects ranged from 0 to 6.83%, indicating varying degrees of genetic interaction. To quantify the mode of action of SNP-SNP interactions, we employed information gain (IGs), which ranged from −8.74% to 3.12%, with the majority (80.21%) of interactions exhibiting negative IG values. This may statistically indicate that these SNPs are involved in overlapping genetic pathways, potentially reflecting functional redundancy in their effects on wood property traits. Moreover, several SNPs within *GRAS50*, including *PtoGRAS50*_SNP960, *PtoGRAS50*_SNP919, *PtoGRAS50*_SNP918, *PtoGRAS50*_SNP998, and *PtoGRAS50*_SNP783, exhibited epistatic effects on holocellulose content (HC), fiber width (FW), FL, pulp yield (PY), and hemicellulose content (HEC) across different loci. Importantly, these results are based on bioinformatic evidence from the natural population and need to be experimentally validated to establish causal biological mechanisms.

### 2.4. Validation of the PtomiR171a-PtoGRAS50 Regulatory Module

**Figure 6:**
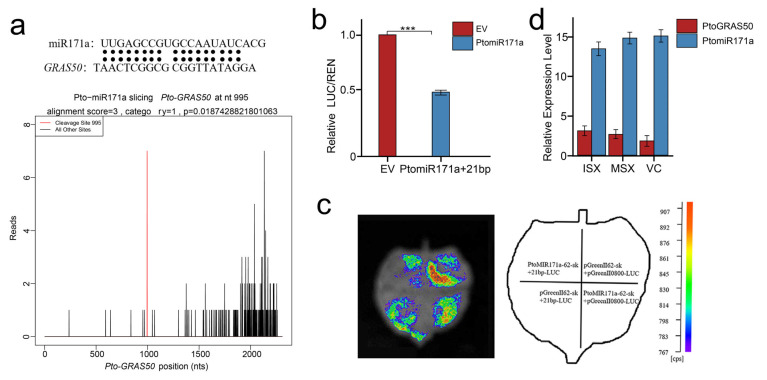
Identification of *GRAS50* as a cleavage target of *PtomiR171a*: (**a**) Schematic representation of the base-pairing interaction between *PtomiR171a* and its target gene *GRAS50*. Degradome sequencing analysis confirms that *miR171a* cleaves *GRAS50*; (**b**,**c**) Luciferase activity analysis validating the interaction between *PtomiR171a* and *PtoGRAS50*. The construct pGreenII 62-SK and pGreenII 62-SK-*PtomiR171a* were used as effectors, while the 21 bp target sequence of *PtoGRAS50* was inserted into the pGreenII 0800-LUC reporter vector. Each value represents the mean  ±  standard deviation (SD) (n  =  3). Asterisks (*p* < 0.05) indicate significant differences compared to the control, *** *p* < 0.001; (**d**) Expression correlation analysis between *PtomiR171a* and *PtoGRAS50* across three tissues, including immature secondary xylem (ISX), mature secondary xylem (MSX), and vascular cambium (VC).

To confirm the potential regulatory interaction between *PtomiR171a* and *PtoGRAS50*, we first performed degradome sequencing to identify miRNA-mediated cleavage sites within *PtoGRAS50* (Figure 6a). This analysis revealed strong evidence for *PtoGRAS50* as a direct target of *miR171a*. To further validate this interaction, we conducted a dual-luciferase reporter assay. The precursor sequence of *PtomiR171a* was cloned into an effector vector, while the 21-bp target sequence of *PtoGRAS50* was inserted into a reporter vector. Co-transformation of pGreenII 62-SK-P*tomiR171a* with pGreenII 0800-LUC-21 bp into *Nicotiana benthamiana* leaves significantly suppressed luciferase activity, reducing the LUC/REN ratio to 0.51 relative to the empty-vector control (pGreenII 62-SK + pGreenII 0800-LUC) (*p* < 0.05) (Figure 6b,c). Additionally, RT-qPCR analysis demonstrated a strong negative correlation (r = −0.76) between *PtomiR171a* and *PtoGRAS50* transcript levels (Figure 6d). *PtomiR171a* was highly expressed in VC but had low expression in ISX, whereas *PtoGRAS50* showed the opposite expression pattern, reinforcing its regulation by *PtomiR171a*. As *PtoGRAS50* is highly expressed during the early stages of xylem development, it likely plays a crucial potential regulatory role in xylem differentiation, fiber elongation, and secondary cell wall formation. Furthermore, the *miR171a*-*GRAS50* module’s association with FL suggests its influence on xylem development and structural integrity. Taken together, these findings provide compelling evidence that *PtoGRAS50* may contribute to fiber development in xylem tissues, with its expression tightly regulated by *PtomiR171a*.

### 2.5. Identification of GRAS50 Target Genes

**Figure 7:**
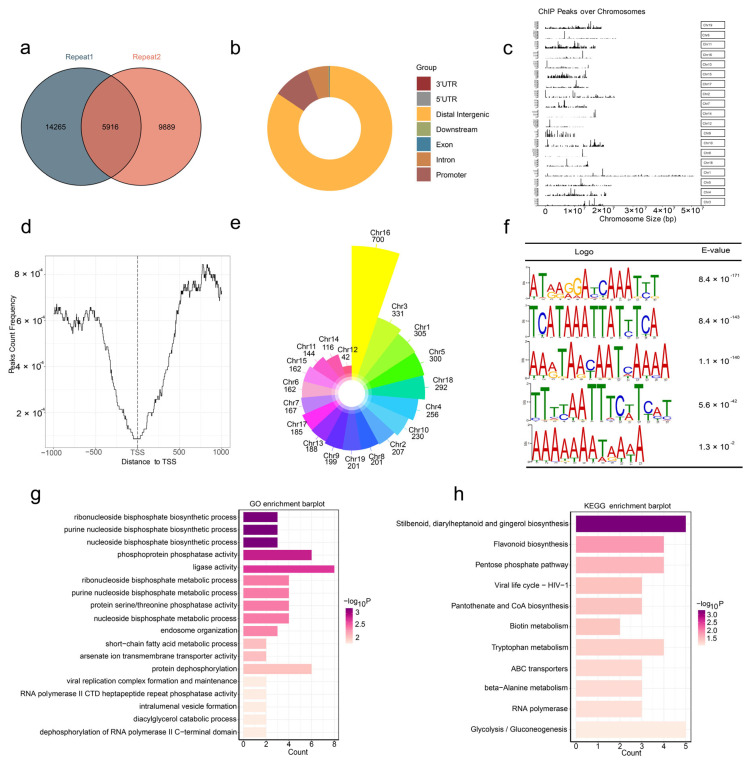
DNA affinity purification sequencing (DAP-seq) analysis of *PtoGRAS50* putative target genes: (**a**) Venn plot showing the overlap of enriched peaks enriched between two biological replicates, indicating reproducibility in *PtoGRAS50* binding site identification; (**b**) Distribution of *PtoGRAS50* binding sites across different genomic regions, including distal intergenic regions, promoter, introns, exons, and UTRs; (**c**) Chromosomal distribution of *GRAS50* binding peaks, highlighting variation in target site density across 19 chromosomes, the Y-axis shows peak frequency calculated as the number of called peaks per 100 kb sliding window; (**d**) DAP-seq signal distribution upstream and downstream of TSS in 1-kb intervals (X-axis), generated using deepTools v3.3.5. The Y-axis represents peak abundance, illustrating binding site enrichment near the promoter region; (**e**) Genome-wide visualization of *PtoGRAS50* binding sites across 19 chromosomes of *P. tomentosa*; (**f**) Conservative motif (E-value ≤ 0.05) enriched in *PtoGRAS50* recognition intervals, suggesting putative cis-regulatory elements involved in gene regulation (**g**) Eighteen significantly enriched GO terms (*p* < 0.05) among genes associated with *PtoGRAS50* binding peaks; (**h**) Eleven significantly enriched KEGG pathways (*p* < 0.05) associated with *PtoGRAS50* putative target genes.

To investigate the potential regulatory targets of *PtoGRAS50*, we conducted DAP-seq, identifying 5016 common peaks across two biological replicates (Figure 7a). These peaks were distributed as follows: 84.48% in the distal intergenic region, 9.72% in the promoter, 5.54% within introns, 0.07% within exons, and 0.02% within 3’-untranslated regions (3’-UTRs) (Figure 7b). Comparative analysis with the reference *P. tomentosa* genome (Figure 7c) revealed that *PtoGRAS50* binding sites were mainly located 500–1000 bp upstream of the transcription start site (TSS) (Figure 7d), suggesting its role in transcriptional regulation. Chromosomal distribution analysis showed that the highest number of binding peaks were identified on chromosomes 16 (700 peaks), 3 (331 peaks), and 1 (305 peaks) (Figure 7e). Further MEME-chip motif analysis identified five significant motifs that likely represent *PtoGRAS50* binding sites within putative target genes: motif1 (ATRAGGATCAAATYT), motif2 (AAAAAAAATAAWA), motif3 (TCATAAATTATTTCA), motif4 (ATCAAAAATAAT), and motif5 (AARTAAYAATCAAAA) (Figure 7f). These motifs provide insights into the cis-regulatory elements recognized by *PtoGRAS50*, further supporting its role in gene regulation during xylem development and fiber formation.

Gene Ontology (GO) analysis revealed that most *PtoGRAS50* putative target genes are associated with nucleotide diphosphate biosynthesis processes and exhibit phosphoprotein phosphatase or general phosphatase activity (Figure 7g). These findings suggest that *PtoGRAS50* may influence cellular signaling and energy metabolism during xylem development. Kyoto Encyclopedia of Genes and Genomes (KEGG) analysis further demonstrated that these putative target genes are primarily involved in stilbenoid, diarylheptanoid, and gingerol biosynthesis, as well as flavonoid biosynthesis (Figure 7h). These metabolic pathways play essential roles in secondary cell wall formation, lignification, and stress responses, indicating potential regulatory functions of *PtoGRAS50* in wood development. Among the identified targets, Ptom.006G.00480 was annotated as participating in cellulose synthase activity, cellulose biosynthetic processes, and cellulose metabolic processes, while Ptom.006G.00230 was associated with the hemicellulose metabolic process. Based on DAP-seq analysis, we identified two key putative target genes, Ptom.006G.00480 and Ptom.006G.00230, which are homologs of *AtCSLE1* and *AtARAF1*, respectively. These genes may play critical roles in xylem development and wood formation in *P. tomentosa*. These findings highlight *PtoGRAS50* as a potential transcriptional regulator of key cell wall biosynthesis genes, further reinforcing its role in fiber development and secondary xylem differentiation.

## 3. Discussion

Tree growth and wood formation are governed by intricate regulatory networks involving multiple genetic components, including TFs and miRNAs. Among these, GRAS family proteins have established roles in meristem maintenance and vascular patterning. In angiosperms, GRAS proteins act as transcriptional cofactors of WUSCHEL and influence downstream programs of cell proliferation and differentiation in vascular and cambial tissues, and their spatial patterns are tightly shaped by miR171-mediated post-transcriptional repression Placing our results in this context, we identified and characterized the PtomiR171 family and its potential regulatory network in *P. tomentosa*, uncovering key interactions that influence wood formation, particularly during the early stage of xylem development. By integrating degradome sequencing, module-based association analysis, and functional validation experiments, we elucidated the PtomiR171a-*PtoGRAS50* regulatory module, which may influence wood properties, particularly fiber length.

### 3.1. Characterization of PtomiR171 and Its Regulatory Network in Populus Tomentosa

miRNAs are indispensable regulators of gene expression, orchestrating various developmental processes across plant species. Among them, miR171 has been extensively studied for its role in regulating shoot architecture, leaf morphology, and chlorophyll accumulation [14]. Despite its identification in numerous species, including *A. thaliana*, *O. sativa*, *Solanum lycopersicum*, *Lilium pumilum*, *Hordeum vulgare*, and *Morus alba* [15], its function in *P. tomentosa* has remained unexplored until now.

We identified 13 members of the PtomiR171 family in *P. tomentosa* and performed comparative analyses with miR171 homologs from five other species. Consistent with observations in other plant species, mature miR171 sequences exhibited higher sequence identity and conservation compared to their precursor sequences (Figure 1c), reinforcing the notion that mature miRNAs are generally more evolutionarily constrained due to their functional importance. Furthermore, we identified conserved sequence motifs such as ‘GAGCCG’ and ‘CAATATC’, which have been previously associated with the HAM branch in *Arabidopsis*. Despite this conservation, both mature and precursor sequences showed some degree of divergence, suggesting functional diversification within the family. Our phylogenetic analysis revealed that *PtomiR171j*, *PtomiR171l*, and *PtomiR171m* clustered with monocot miR171 members, suggesting potential functional divergence within *P. tomentosa*. This aligns with previous findings in other plant species, where miR171 has evolved to regulate distinct biological processes [16]. Overall, these findings reflect a complex evolutionary history, combining ancient conserved functions with recent lineage-specific diversification, shedding light on the regulatory significance of miR171 in *P. tomentosa*.

Understanding gene expression patterns is fundamental to elucidating their biological functions, as gene activity is often tightly linked to developmental processes and environmental responses. In plants, miRNA genes are typically expressed in a spatiotemporally regulated manner, allowing them to fine-tune gene expression at the post-transcriptional level. The expression patterns of the miR171 family have been extensively studied in various species using confocal imaging of fluorescent reporters and in situ hybridization [17]. Applying similar approaches to *P. tomentosa* could provide a more detailed understanding of PtomiR171 expression dynamics, particularly in relation to wood formation. Our expression analysis revealed that *PtomiR171a* and *PtomiR171k* are highly expressed in VC and MSX, suggesting their potential roles in wood formation (Figure 1d). Furthermore, the strong conservation of *PtomiR171a* across species reinforces its functional importance. As a result, *PtomiR171a* was selected for further investigation in this study. A previous study has demonstrated that miR171 is responsive to environmental signals, including light and abiotic stresses [18]. Further research should focus on elucidating the molecular mechanisms by which miR171 integrates environmental factors into plant development processes, providing deeper insights into its regulatory roles in stress adaptation and growth modulation.

### 3.2. Allelic Variations in the PtomiR171-Target Module Affect Wood Formation in Populus

The validation of miRNA target genes has been widely conducted using degradome sequencing across various plant species [19,20]. In this study, we used degradome data to construct a miR171 regulatory network and identified six putative targets from the GRAS and DHHC families. Although our downstream analyses focused on the Pto*miR171a*-*PtoGRAS50* module because it showed the strongest association with FL and was supported by DEG in relation to early stages of xylem development, miR171a likely acts within a broader regulatory network. Other identified targets may have direct or indirect effects on wood traits, and combinations of allelic variation across multiple targets could possibly contribute to the association signals observed in the natural population.

Candidate gene association analysis has proven useful for discovering genetic variants related to wood traits in forest trees and *P. tomentosa* [21,22], and our results similarly nominate *PtomiR171a-PtoGRAS50* as a promising candidate influencing FL. To reduce confounding from population structure and relatedness, we applied a mixed linear model (MLM) including Q and K matrices and performed FDR correction; we also inspected pairwise LD when prioritizing SNP-SNP pairs. Nevertheless, residual LD or pleiotropy may still affect association signals. Notably, most significant SNP-SNP pairs showed negative interaction information (ΔIG < 0), which can reflect redundancy or shared information among loci arising from functional overlap or phenotypic noise. These results, therefore, point to complex functional relationships within the PtomiR171-GRAS module.

In summary, while our degradome, association and functional assays support a regulatory role for *PtomiR171a-PtoGRAS50* in xylem development and FL, definitive attribution of phenotypic effects to this single target versus multiple targets will require targeted experiments. Some follow-up approaches, such as allele-specific expression analyses, combinatorial knockdown/overexpression, or targeted Clustered Regularly Interspaced Short Palindromic Repeats (CRISPR) allelic swaps, would be applied to disentangle individual, additive or epistatic contributions of other PtomiR171 targets and to validate causal variants.

### 3.3. Interaction Within the PtomiR171a-PtoGRAS50 Regulatory Cascade

The intricate regulatory roles of miRNAs in plant development, particularly in wood formation, are increasingly recognized. In this study, we focused on the miR171 family in *P. tomentosa* and validated the interaction between *PtomiR171a* and its target *PtoGRAS50* using a dual luciferase assay. This assay confirmed that PtomiR171a cleaves a specific 21-nt target sequence within *PtoGRAS50*, demonstrating the reliability of dual luciferase assays as a robust tool for investigating miRNA-target interactions. Further supporting this interaction, expression analyses revealed a significant negative correlation between *PtomiR171a* and *PtoGRAS50* across multiple tissues. This suggests that PtomiR171a functions as a negative regulator of *PtoGRAS50*, consistent with the general mechanism of miRNA-mediated gene suppression through mRNA cleavage or translational inhibition. To further substantiate these findings, additional experimental approaches could be employed. 5’-rapid amplification of cDNA ends (5’-RACE) is a powerful tool that could precisely verify the binding site of miRNA and its target. Additionally, β-glucuronidase (GUS) staining assays could be used to validate translational inhibition of miRNAs on their targets [23]. Thus, further molecular experiments and transgenic strategies to confirm the interaction between miR171 family members and their targets in *P. tomentosa* would provide deeper insights into their functional significance in xylem development and wood formation, strengthening our understanding of miRNA-based regulatory networks in trees.

Beyond its direct interaction with *PtomiR171a*, we identified that *PtoGRAS50* may have important functions during the early xylem development stage and further influence fiber development by binding to the promoter regions of two downstream genes. However, this conclusion still needs to be verified by transgenic strategies in the future, and the internal control in the RT-qPCR of *GRAS50* was not so common in plants, and its stability needs to be empirically confirmed in the studied tissues and at different developmental stages in the future. Ptom.006G.00480 and Ptom.006G.00230, homologs of Cellulose synthase-like E1 (*AtCSLE1*) (AT1G55850) and α-l-arabinofuranosidase (*AtARAF1*) (AT3G10740), respectively, were annotated with roles in cellulose and hemicellulose metabolism [24,25]. Although these regulatory links require confirmation by transgenic and in vivo binding assays, they refine the putative *PtomiR171a-PtoGRAS50* cascade and provide practical entry points for wood-quality improvement in *Populus*. For instance, modulation of *GRAS50* or its downstream targets via overexpression or CRISPR/Cas-based editing could be used to generate *Populus* genotypes with improved wood properties.

## 4. Materials and Methods

### 4.1. Association Population and Phenotypic Data

The association population in this study comprised 303 unrelated *P. tomentosa* individuals, selected from a broader collection of 1047 trees representing the species’ natural distribution across China. The population was originally planted in 1982 in Guan Xian County, Shandong Province (36°23′ N, 115°47′ E), following a completely randomized block design with three clonal replicates to ensure robust statistical analysis.

Eight wood traits were evaluated for association analysis, including fiber width (FW, mm), α-cellulose content (CC, %), hemicellulose content (HEC, %), pulp yield (PY, %), lignin content (LC, %), holocellulose content (HC, %), microfibril (MFA, °), and FL (mm). Each trait was measured using three biological replicates. Detailed methodologies for trait measurement and phenotypic variation were previously described by Du et al. [26].

### 4.2. The Single-Nucleotide Polymorphism (SNP) Calling and Association Analysis

The 303 unrelated individuals of *P. tomentosa* were resequenced using the Illumina GA II platform (Shanghai, China) with a depth of >30 × (raw data). We used VCFtools v0.1.16 to extract gene-derived biallelic SNPs from 13 PtomiR171 family members and six putative target genes, including 1000 bp flanking sequences of Pri-miR171 and 2000 bp flanking sequences of putative target genes. A total of 1843 high-quality SNPs were identified within these candidate genomic regions, applying a threshold of MAF > 5% and a missing data rate < 20% across the 303 accessions.

Candidate gene association analysis was performed using TASSEL v5.0 with a mixed linear model (MLM) implementing the Q + K framework, as previously described [27]. Population structure (Q) was inferred by principal component analysis (PCA) on genome-wide marker genotypes and the top principal components were included as covariates. Kinship (K) was estimated from the same marker set using the centered identity-by-state method in TASSEL to generate a pairwise relatedness matrix. The Q + K MLM thus controls for both population stratification and relatedness. Multiple testing was corrected using the QVALUE v2.36.0 package (FDR), and SNPs with Q < 0.1 (FDR) were considered significant.

Multifactor Dimensionality Reduction (MDR) v3.0.2 software was employed to detect epistatic interactions among the identified SNPs. To enhance the reliability of probability approximation, all unlinked SNPs were filtered using the default settings. An entro-py-based measure was used to detect significant SNP-SNP interactions, while IG scores were calculated to evaluate epistatic effects. All *p* values were corrected by using the Benjamini-Hochberg methods.

### 4.3. Differential Gene Expression Analysis

Total RNA was extracted from the xylem of *P. tomentosa* clone ‘LM50’, sampled at different developmental stages (1, 2, 3, 4, 5, and 10 years old) from trees planted in Guan Xian County. Three biological replicates were used per developmental stage to ensure statistical robustness. Strand-specific RNA-seq libraries were constructed and sequenced using the Illumina HiSeq X10 platform, with all library preparation and sequencing performed by NovoGene Co., Ltd. (Beijing, China). For data preprocessing, raw reads underwent quality control and adapter trimming using fastp v0.23.4. The resulting clean reads were then aligned to the *P. tomentosa* genome using HISAT2 v2.1.0 with default parameters. Following alignment, transcript assembly and quantification were conducted using StringTie v2.1.7, enabling accurate measurement of gene expression across different developmental stages.

Read counts were normalized, and DEGs were identified using DESeq2 v1.40.2. A stringent significance threshold was applied, where DEGs were defined by |Log2 (fold change) | > 1.0 and FDR < 0.05. To illustrate the overlap of DEGs across different development stages, Venn diagrams were generated using VENNY v2.1. Additionally, DEG visualization was performed using the EnhancedVolcano package in R v3.4.1.

### 4.4. Identification and Analysis of PtomiR171 and Target Sequences

To identify miR171 family members in *P. tomentosa*, a total of 13 miR171 sequences from *P. trichocarpa* were retrieved from the miRbase database. These sequences were used as queries for homology searches using the Basic Local Alignment Search Tool (BLAST v2.17.0) (http://blast.ncbi.nlm.nih.gov/) (accessed on 14 December 2025), leading to the identification of an equivalent set of 13 homologous sequences in *P. tomentosa*, which were subsequently named PtomiR171s. The physical position and chromosomal distributions of PtomiR171 family members were determined through BLAST searches and graphically visualized using TBtools v2.155 software.

To explore the evolutionary relationships of the PtomiR171 family, a phylogenetic tree was constructed using precursor sequences from both dicotyledonous and monocotyledonous plants. The dataset included 13 PtomiR171s and three miR171 members from A. thaliana, nine from *Oryza sativa*, two from *Triticum aestivum*, and 13 from *Zea mays*. The tree was generated using the neighbor-joining (NJ) method with 1000 bootstrap replicates and visualized using Evolview (http://www.evolgenius.info/evolview/) (accessed on 14 December 2025). Multiple sequence alignment was performed using the ClustalW program in MEGA-X, with the alignment results submitted to ESPript3 (https://espript.ibcp.fr/ESPript/ESPript/index.php) (accessed on 14 December 2025) for visualization. Additionally, RNA secondary structure prediction was conducted for PtomiR171 precursor sequences using RNAfold (http://rna.tbi.univie.ac.at/cgi-bin/RNAWebSuite/RNAfold.cgi) (accessed on 14 December 2025), with default parameters applied to calculate the minimum free energy (MFE), providing insights into the structural stability of these miRNAs.

The putative target genes of PtomiR171 were identified using degradome sequencing, a technique that enables the precise detection of miRNA-mediated cleavage sites by sequencing RNA 5’ ends. This approach provides direct evidence of miRNA-target interactions by capturing the degradation products of cleaved transcripts. The degradome-sequencing procedure was performed following previously established protocols [28]. Alignments with scores up to four, where G:U pairs scored 0.5 and no mismatches were found at the site between the 10th and 11th nucleotides of the corresponding miRNAs, were considered potential targets.

### 4.5. Quantitative Reverse Transcriptase Polymerase Chain Reaction

To evaluate the expression of PtomiR171 and its putative target genes, eight distinct tissues were collected from a single *P. tomentosa* ‘LM50’ plant, including MSX, ISX, vascular cambium VC, SP, BK, mature leaf (ML), root (RT), and apex (AP). Immediately after collection, samples were flash-frozen in liquid nitrogen. Total RNA was extracted from each sample (three biological replicates per sample) using the FastPure Plant Total RNA Isolation Kit (Vazyme, Nanjing, China), following the manufacturer’s protocol. First-strand complementary DNA (cDNA) was synthesized using the Reverse Transcription System (Promega Corporation, Madison, WI, USA) for mRNA transcripts and the miRcute miRNA First-Strand cDNA Synthesis Kit (Tiangen, Beijing, China) for miRNA-specific reverse transcription.

Quantitative reverse transcriptase polymerase chain reaction (RT-qPCR) was performed using the 3500 Fast RT-PCR system (Thermo Fisher, Waltham, MA, USA) with SYBR premix Ex Taq (TaKaRa, Tokyo, Japan), following the manufacturer’s instructions. Gene-specific primers were designed using Primer Express 5.0 (Thermo Fisher, Waltham, MA, USA) and are listed in Table S4. 5.8S rRNA and 18S rRNA were used as the internal reference genes for miRNA and mRNA normalization, respectively. For target gene analysis, RT-qPCR conditions included an initial preincubation at 95 °C for 30 s, followed by 40 cycles of 95 °C for 5 s and 60 °C for 5 s. For the analysis of PtomiR171 mature sequences, the reaction began with a preincubation at 95 °C for 15 min, followed by 40 cycles of 94 °C for 20 s and 60 °C for 34 s. Automated melting curve analysis was used to verify specificity. The relative transcript abundance of candidate genes was quantified using the 2−ΔΔCt method. All RT-qPCR reactions were performed in duplicate across a minimum of three biological replicates, ensuring data reliability and reproducibility.

### 4.6. Dual-Luciferase Reporter Assay

To validate the regulatory interaction between *PtomiR171a* and its target gene *PtoGRAS50*, precursor sequences of *PtomiR171a* and the 21-bp target sequence of *PtoGRAS50* were amplified from 1-year-old *P. tomentosa* ‘LM50’ clone. The primers used for amplification are listed in Table S4. The amplified *PtomiR171a* precursor sequence was cloned into the effector plasmid (pGreenII 62-SK vector), while the *PtoGRAS50* target sequence was inserted into the reporter plasmid (pGreenII 0800-LUC vector). The effector and reporter constructs were then co-infiltrated into *N*. *benthamiana* leaves for transient expression. Fluorescence imaging of infiltrated leaves was captured using the LB985 NightSHADE fluorescence imaging system (Berthold Technologies, Shanghai, China). Dual-luciferase activity was measured using a GloMax 20/20 luminometer (Promega Corporation, Madison, WI, USA) and a Dual-Luciferase Assay Kit (Promega Corporation, Madison, WI, USA), following the manufacturer’s instructions. All experiments were performed at least three times as independent biological replicates.

### 4.7. DNA Affinity Purification, Sequencing and Enrichment Analyses

DAP-seq was performed at Bluelanscape Hebei Biotechnology (Shijiazhuang, Hebei, China). The coding sequence (CDS) of *PtoGRAS50* was cloned into the pFN19K HaloTag T7 SP6 Flexi vector (Promega Corporation, Madison, WI, USA) and expressed using the TNT SP6 Coupled Wheat Germ Extract System (Promega Corporation, Madison, WI, USA). Genomic DNA (gDNA) was extracted from leaves of *P. tomentosa* clone ‘LM50’ and fragmented by sonication to generate 200–800 bp fragments. The recombinant Halo-*PtoGRAS50* fusion protein was immobilized onto anti-Halo monoclonal antibody agarose beads (Promega Corporation, Madison, WI, USA) and incubated with 200 ng of fragmented gDNA for 1 h at room temperature. Following incubation, the beads were washed to remove unbound DNA, and the bound DNA fragments were recovered for sequencing. The enriched DNA libraries were pooled and sequenced using the Illumina Novaseq 6000 platform (Beijing, China), generating 150-bp paired-end reads. Input DNA libraries were prepared as described above to control for background noise.

DAP-seq reads were aligned to the reference *P. tomentosa* genome using Bowtie2 v2.5.4. To ensure high-confidence mapping, SAMtools v1.19.2 was used to filter mapped reads, retaining only uniquely mapped reads for downstream analyses. Peak calling was performed using Model-based Analysis for ChIP-Seq (MACS2) v2 to identify significant DNA-binding regions of *PtoGRAS50*. DAP-seq peaks located within 2 kb upstream or downstream of the TSS were annotated using BEDtools v2.31.1 based on General Feature Format (GFF) files. For data visualization, ChIPseeker v1.22.1 was employed to generate peak coverage maps across chromosomes to profile binding sites within TSS regions. Conserved motif discovery was conducted using the MEME-ChIP (https://meme-suite.org/meme/doc/meme-chip.html) (accessed on 14 December 2025) suite to identify potential regulatory motifs within the enriched sequences. To investigate the functional significance of *PtoGRAS50* putative target genes, GO and KEGG pathway analyses were performed using the KOBAS v2.0 database (http://bioinfo.org/kobas/) (accessed on 14 December 2025).

## 5. Conclusions

In summary, our study elucidates the functional conservation and diversity within the PtomiR171 family and investigates the genetic effects of allelic variants in PtomiR171 and its target gene modules using molecular biology and association genetics approaches. Through functional validation and regulatory network analysis, we refine the potential *PtomiR171a*-*PtoGRAS50* cascade, shedding light on its role in wood formation. These findings not only enhance our understanding of miRNA-mediated regulatory mechanisms in *P. tomentosa* but also highlight *PtomiR171a*-*PtoGRAS50* as a key genetic module with potential applications in breeding programs aimed at improving wood properties.

## Figures and Tables

**Table 1:** Summary of significant SNPs with the PtomiR171 family and the putative target genes associated with wood properties in the association population of *P. tomentosa.*

Traits	Gene ID	Chr	Position	*p*-Value	*Q*-Value
FL	*PtoGRAS50*	14	3740492	3.69 × 10^−4^	9.04 × 10^−2^
FL	*PtomiR171l*	15	523018	3.69 × 10^−4^	9.04 × 10^−2^
FL	*PtomiR171f*	2	3695682	2.54 × 10^−4^	7.87 × 10^−2^
FL	*PtoGRAS14*	2	11031528	2.76 × 10^−4^	7.87 × 10^−2^
FL	*PtoGRAS14*	2	11031564	2.76 × 10^−4^	7.87 × 10^−2^
FL	*PtomiR171a*	4	17508135	2.41 × 10^−4^	9.04 × 10^−2^
HEC	*PtomiR171m*	12	4951111	1.01 × 10^−4^	7.44 × 10^−2^

## Data Availability

The raw data from the genome resequencing of the 303 *Populus tomentosa* accessions and all RNA-seq data for the differently aged *P. tomentosa* clones have been submitted to the Genome Sequence Archive in the BIG Data Center, Beijing Institute of Genomics (BIG), Chinese Academy of Sciences (CAS) under accession numbers CRA000903 and CRA004084. Both data sets are publicly accessible at (http://bigd.big.ac.cn/gsa/) (accessed on 14 December 2025). The reference genome of *P. tomentosa* was retrieved from CNGB Sequence Archive (CNSA) of China National GeneBank DataBase (CNGBdb) with CNSA project ID CNP0004290, and is publicly accessible at https://db.cngb.org/.

## Supplementary Materials

The following supporting information can be downloaded at: https://www.mdpi.com/article/10.3390/ijms27010228/s1.

## Abbreviations

The following abbreviations are used in this manuscript:

AP	Apex
BK	Bark
CC	α-cellulose content
CLV3	CLAVATA3
DAP-seq	DNA affinity purification sequencing
FL	Fiber length
FW	Fiber width
GFF	General feature format
GO	Gene ontology
GWAS	Genome-wide association studies
HAM	Hairy meristem
HC	Holocellulose content
HEC	Hemicellulose content
IG	Information gain
ISX	Immature secondary xylem
JW	Juvenile wood
KEGG	Kyoto encyclopedia of genes and genomes
LC	Lignin content
MACS2	Model-based analysis for ChIP-Seq
MAF	Minor allele frequency
MDR	Multifactor dimensionality reduction
MFA	Microfibril angle
miRNAs	microRNAs
ML	Mature leaf
MLM	Mixed linear model
MSX	Mature secondary xylem
MW	Mature wood
NSP2	Nodulation signaling pathway2
nt	Nucleotides
pri-miRNAs	Primary microRNAs
PY	Pulp yield
RNA-seq	RNA sequencing
RT	Root
SCL	Scarecrow-like
SP	Secondary phloem
sRNA-seq	SmallRNA sequencing
TFs	Transcription factors
TSS	Transcription start site
VC	Vascular cambium
WUS	WUSCHEL

## References

[1] Guan X., Pang M., Nah G., Shi X., Ye W., Stelly D.M., Chen Z.J. (2014). miR828 and miR858 regulate homoeologous MYB2 gene functions in Arabidopsis trichome and cotton fibre development. Nat. Commun..

[2] Zhang B., Liu G., Song J., Jia B., Yang S., Ma J., Liu J., Shahzad K., Wang W., Pei W. (2022). Analysis of the MIR396 gene family and the role of MIR396b in regulating fiber length in cotton. Physiol. Plant..

[3] Zhou Y., Yan A., Han H., Li T., Geng Y., Liu X., Meyerowitz E.M. (2018). HAIRY MERISTEM with WUSCHEL confines CLAVATA3 expression to the outer apical meristem layers. Science.

[4] Devers E.A., Branscheid A., May P., Krajinski F. (2011). Stars and symbiosis: microRNA-and microRNA*-mediated transcript cleavage involved in arbuscular mycorrhizal symbiosis. Plant Physiol..

[5] Lauressergues D., Delaux P.M., Formey D., Lelandais-Brière C., Fort S., Cottaz S., Bécard G., Niebel A., Roux C., Combier J.P. (2012). The microRNA miR171h modulates arbuscular mycorrhizal colonization of Medicago truncatula by targeting NSP2. Plant J..

[6] Hendelman A., Kravchik M., Stav R., Frank W., Arazi T. (2016). Tomato HAIRY MERISTEM genes are involved in meristem maintenance and compound leaf morphogenesis. J. Exp. Bot..

[7] Sharma R., Draicchio F., Bull H., Herzig P., Maurer A., Pillen K., Thomas W.T.B., Flavell A.J. (2018). Genome-wide association of yield traits in a nested association mapping population of barley reveals new gene diversity for future breeding. J. Exp. Bot..

[8] Xu P., Zhang W., Wang X., Zhu Y., Liang W., He Y., Yu X. (2023). Multiomics analysis reveals a link between Brassica-specific miR1885 and rapeseed tolerance to low temperature. Plant Cell Environ..

[9] Carré C., Carluer J.B., Chaux C., Estoup-Streiff C., Roche N., Hosy E., Mas A., Krouk G. (2024). Next-Gen GWAS: Full 2D epistatic interaction maps retrieve part of missing heritability and improve phenotypic prediction. Genome Biol..

[10] Chen J., Xie J., Chen B., Quan M., Li Y., Li B., Zhang D. (2016). Genetic variations and miRNA-target interactions contribute to natural phenotypic variations in Populus. New Phytol..

[11] Wang Y., Luo J., Zhang H., Lu J. (2016). microRNAs in the Same Clusters Evolve to Coordinately Regulate Functionally Related Genes. Mol. Biol. Evol..

[12] Pei L.L., Zhang L.L., Liu X., Jiang J. (2023). Role of microRNA miR171 in plant development. PeerJ.

[13] Barrios A., Trincado G., Watt M.S. (2016). Wood Properties of Juvenile and Mature Wood of Pinus radiata D. Don Trees Growing on Contrasting Sites in Chile. For. Sci..

[14] Schulze S., Schäfer B.N., Parizotto E.A., Voinnet O., Theres K. (2010). LOST MERISTEMS genes regulate cell differentiation of central zone descendants in Arabidopsis shoot meristems. Plant J..

[15] Yan R., Song S., Li H., Sun H. (2022). Functional analysis of the eTM-miR171-SCL6 module regulating somatic embryogenesis in Lilium pumilum DC. Fisch. Hortic. Res..

[16] Engstrom E.M., Andersen C.M., Gumulak-Smith J., Hu J., Orlova E., Sozzani R., Bowman J.L. (2011). Arabidopsis homologs of the petunia hairy meristem gene are required for maintenance of shoot and root indeterminacy. Plant Physiol..

[17] Han H., Yan A., Li L., Zhu Y., Feng B., Liu X., Zhou Y. (2020). A signal cascade originated from epidermis defines apical-basal patterning of Arabidopsis shoot apical meristems. Nat. Commun..

[18] Siré C., Moreno A.B., Garcia-Chapa M., López-Moya J.J., San Segundo B. (2009). Diurnal oscillation in the accumulation of Arabidopsis microRNAs, miR167, miR168, miR171 and miR398. FEBS Lett..

[19] Llave C., Xie Z., Kasschau K.D., Carrington J.C. (2002). Cleavage of Scarecrow-like mRNA targets directed by a class of Arabidopsis miRNA. Science.

[20] Geng Y., Xie C., Zhang C., Liu X., Zhou Y. (2025). Functions and Regulation of HAM Family Genes in Meristems During Gametophyte and Sporophyte Generations. Plant Cell Environ..

[21] Beaulieu J., Nadeau S., Ding C., Celedon J.M., Azaiez A., Ritland C., Laverdière J.P., Deslauriers M., Adams G., Fullarton M. (2020). Genomic selection for resistance to spruce budworm in white spruce and relationships with growth and wood quality traits. Evol. Appl..

[22] Du Q., Tian J., Yang X., Pan W., Xu B., Li B., Ingvarsson P.K., Zhang D. (2015). Identification of additive, dominant, and epistatic variation conferred by key genes in cellulose biosynthesis pathway in Populus tomentosa. DNA Res. Int. J. Rapid Publ. Rep. Genes Genomes.

[23] Gong J., Tong Y., Zhang H.M., Wang K., Hu T., Shan G., Sun J., Guo A.Y. (2012). Genome-wide identification of SNPs in microRNA genes and the SNP effects on microRNA target binding and biogenesis. Hum. Mutat..

[24] Chávez Montes R.A., Ranocha P., Martinez Y., Minic Z., Jouanin L., Marquis M., Saulnier L., Fulton L.M., Cobbett C.S., Bitton F. (2008). Cell wall modifications in Arabidopsis plants with altered alpha-L-arabinofuranosidase activity. Plant Physiol..

[25] Jung S.E., Bang S.W., Kim S.H., Seo J.S., Yoon H.B., Kim Y.S., Kim J.K. (2021). Overexpression of OsERF83, a Vascular Tissue-Specific Transcription Factor Gene, Confers Drought Tolerance in Rice. Int. J. Mol. Sci..

[26] Du Q., Xu B., Pan W., Gong C., Wang Q., Tian J., Li B., Zhang D. (2013). Allelic variation in a cellulose synthase gene (PtoCesA4) associated with growth and wood properties in Populus tomentosa. G3 Genes Genomes Genet..

[27] Du Q., Yang X., Xie J., Quan M., Xiao L., Lu W., Tian J., Gong C., Chen J., Li B. (2019). Time-specific and pleiotropic quantitative trait loci coordinately modulate stem growth in Populus. Plant Biotechnol. J..

[28] Xiao L., Quan M., Du Q., Chen J., Xie J., Zhang D. (2017). Allelic Interactions among Pto-MIR475b and Its Four Target Genes Potentially Affect Growth and Wood Properties in Populus. Front. Plant Sci..

