# Supplementary material for: Identification of miR171a-GRAS50 Regulatory Module Associated with Wood Properties in Populus tomentosa
Source: Int J Mol Sci. 2025 Dec 25;27(1):228. doi: 10.3390/ijms27010228 (PMC12785576; doi:10.3390/ijms27010228)
Supplement: Supplementary file 1 [file ijms-27-00228-s001.zip › ijms-4018876-supplementary.pdf]

Identification of miR171a-*GRAS50* regulatory module associated with wood properties in *Populus tomentosa*

Guhang Shi[1, 2], Rui Huang[1, 2], Shitong Qin[1, 2], Mingyang Quan[1, 2] and Deqiang Zhang[1, 2*]

[1]State Key Laboratory of Tree Genetics and Breeding, College of Biological Sciences and Technology, Beijing Forestry University, Beijing, 100083, China.

[2]National Engineering Research Center of Tree Breeding and Ecological Restoration, College of Biological Sciences and Technology, Beijing Forestry University, Beijing, 100083, China.

* Correspondence: Deqiang Zhang, E-mail: deqiangzhang@bjfu.edu.cn.

SUPPLEMENTARY TABLES

The contents included 4 tables:

Table S1. miR171 family and its target gene location in *Populus tomentosa*

Table S2. Results of Degradome sequencing

Table S3. SNP pairs and their main effects in *Populus tomentosa* association population

Table S4. Gene-specific primers used in the present study

Table S1. miR171 family and its target gene location in *Populus tomentosa*

miRNA(precursor sequences)	Chr	Start-position	End-position	Length
PtomiR171a	Chr4	17507992	17508095	103
PtomiR171b	Chr11	6190431	6190526	95
PtomiR171c	Chr6	4789106	4789209	103
PtomiR171d	Chr18	7880668	7880767	99
PtomiR171e	Chr1	33668763	33668856	93
PtomiR171f	Chr2	937607	937736	129
PtomiR171g	Chr12	10870625	10870724	99
PtomiR171h	Chr15	11187640	11187739	99
PtomiR171i	Chr5	8073955	8074080	125
PtomiR171j	Chr12	4958131	4958246	115
PtomiR171k	Chr15	3542363	3542440	77
PtomiR171l	Chr15	522450	522556	106
PtomiR171m	Chr12	4951260	4951360	100

Gene ID	Chr	Start-position	End-position	Length
PtoGRAS3	Chr1	10701798	10705869	4071
PtoGRAS14	Chr2	11032449	11035911	3462
PtoGRAS15	Chr2	11503981	11507381	3400
PtoGRAS49	Chr14	3695741	3698049	2308
PtoGRAS50	Chr14	3736091	3738849	2758

Table S2. Results of Degradome sequencing

miRNA	Target	Annotation	Gene ID	Best_blast_Ptri
PtomiR171a	PtoGRAS14	GRAS family transcription factor	Ptom.002G.015781	Potri.002G144200
PtomiR171a	PtoGRAS15	GRAS family transcription factor	Ptom.002G.01629	Potri.002G144700
PtomiR171a	PtoGRAS49	GRAS family transcription factor	Ptom.014G.00443	Potri.014G060200
PtomiR171a	PtoGRAS50	GRAS family transcription factor	Ptom.014G.004441	Potri.014G060500
PtomiR171b	PtoGRAS14	GRAS family transcription factor	Ptom.002G.015781	Potri.002G144200
PtomiR171b	PtoGRAS15	GRAS family transcription factor	Ptom.002G.01629	Potri.002G144700
PtomiR171b	PtoGRAS49	GRAS family transcription factor	Ptom.014G.00443	Potri.014G060200
PtomiR171b	PtoGRAS50	GRAS family transcription factor	Ptom.014G.004441	Potri.014G060500
PtomiR171c	PtoGRAS3	GRAS family transcription factor	Ptom.001G.01188	Potri.001G122800
PtomiR171c	PtoGRAS49	GRAS family transcription factor	Ptom.014G.00443	Potri.014G060200
PtomiR171d	PtoGRAS3	GRAS family transcription factor	Ptom.001G.01188	Potri.001G122800
PtomiR171d	PtoGRAS49	GRAS family transcription factor	Ptom.014G.00443	Potri.014G060200
PtomiR171e	PtoGRAS3	GRAS family transcription factor	Ptom.001G.01188	Potri.001G122800
PtomiR171e	PtoGRAS49	GRAS family transcription factor	Ptom.014G.00443	Potri.014G060200
PtomiR171f	PtoGRAS3	GRAS family transcription factor	Ptom.001G.01188	Potri.001G122800
PtomiR171f	PtoGRAS49	GRAS family transcription factor	Ptom.014G.00443	Potri.014G060200
PtomiR171g	PtoGRAS3	GRAS family transcription factor	Ptom.001G.01188	Potri.001G122800
PtomiR171g	PtoGRAS49	GRAS family transcription factor	Ptom.014G.00443	Potri.014G060200
PtomiR171h	PtoGRAS3	GRAS family transcription factor	Ptom.001G.01188	Potri.001G122800
PtomiR171h	PtoGRAS49	GRAS family transcription factor	Ptom.014G.00443	Potri.014G060200
PtomiR171i	PtoGRAS3	GRAS family transcription factor	Ptom.001G.01188	Potri.001G122800
PtomiR171i	PtoGRAS49	GRAS family transcription factor	Ptom.014G.00443	Potri.014G060200
PtomiR171j	PtoDHHC14	DHHC-type zinc finger family protein	Ptom.002G.01655	Potri.002G147300
PtomiR171k	PtoGRAS3	GRAS family transcription factor	Ptom.001G.01188	Potri.001G122800
PtomiR171k	PtoGRAS49	GRAS family transcription factor	Ptom.014G.00443	Potri.014G060200
PtomiR171l	PtoDHHC14	DHHC-type zinc finger family protein	Ptom.002G.01655	Potri.002G147300
PtomiR171m	PtoDHHC14	DHHC-type zinc finger family protein	Ptom.002G.01655	Potri.002G147300

Table S3. SNP pairs and their main effects in *Populus tomentosa* association population

Traits	Attribute A	Position	Attribute B	Position	Effect of interaction	Information gain
Angle	Ml-11	15_522841	Mb-2	11_6189944	1.0025	-0.0468
Angle	Mf-6	2_938256	Mb-2	11_6189944	0.1234	-0.0105
Angle	Mf-6	2_938256	Ml-11	15_522841	0.2607	-0.0046
Angle	Mg-7	12_10871340	Mb-2	11_6189944	0.1914	-0.0237
Angle	Mg-7	12_10871340	Ml-11	15_522841	0.8628	-0.0268
Angle	Mg-7	12_10871340	Mf-6	2_938256	0.1594	-0.0229
Angle	T49-17	14_3695682	Mb-2	11_6189944	0.1234	-0.0105
Angle	T49-17	14_3695682	Ml-11	15_522841	0.2607	-0.0046
Angle	T49-17	14_3695682	Mf-6	2_938256	0.0235	0.0002
Angle	T49-17	14_3695682	Mg-7	12_10871340	0.1594	-0.0229
Angle	T49-19	14_3700175	Mb-2	11_6189944	0.4492	-0.0695
Angle	T49-19	14_3700175	Ml-11	15_522841	1.4018	-0.0571
Angle	T49-19	14_3700175	Mf-6	2_938256	0.8392	0.0003
Angle	T49-19	14_3700175	Mg-7	12_10871340	0.9602	-0.0232
Angle	T49-19	14_3700175	T49-17	14_3695682	0.8392	0.0003
α-cellulose	Ml-12	15_523400	Mk-10	15_3541642	0.0324	-0.0324
α-cellulose	Mm-14	12_4952199	Mk-10	15_3541642	0.2644	-0.0117
α-cellulose	Mm-14	12_4952199	Ml-12	15_523400	0.1965	-0.0384
α-cellulose	T49-18	14_3700102	Mk-10	15_3541642	0.2183	-0.0278
α-cellulose	T49-18	14_3700102	Ml-12	15_523400	0.0914	-0.0113
α-cellulose	T49-18	14_3700102	Mm-14	12_4952199	0.4354	-0.0176
α-cellulose	T50-28	14_3739726	Mk-10	15_3541642	0.5348	-0.0863
α-cellulose	T50-28	14_3739726	Ml-12	15_523400	0.4669	-0.0732
α-cellulose	T50-28	14_3739726	Mm-14	12_4952199	0.684	-0.0874
α-cellulose	T50-28	14_3739726	T49-18	14_3700102	0.5258	-0.0537
α-cellulose	T3-16	1_10702624	Mk-10	15_3541642	0.2124	-0.0219
α-cellulose	T3-16	1_10702624	Ml-12	15_523400	0.1445	-0.008
α-cellulose	T3-16	1_10702624	Mm-14	12_4952199	0.4295	-0.0025
α-cellulose	T3-16	1_10702624	T49-18	14_3700102	0.2714	0.0312
α-cellulose	T3-16	1_10702624	T50-28	14_3739726	0.5199	-0.0386
Fiber length	Mk-8	15_3541748	Mb-3	11_6189589	0.1594	0
Fiber length	Mk-9	15_3543339	Mb-3	11_6189589	0.1594	0.0244
Fiber length	Mk-9	15_3543339	Mk-8	15_3541748	0.1594	0
Fiber length	Ml-13	15_521982	Mb-3	11_6189589	0.1914	0
Fiber length	Ml-13	15_521982	Mk-8	15_3541748	0.2504	-0.037
Fiber length	Ml-13	15_521982	Mk-9	15_3543339	0.1914	0
Fiber length	T49-20	14_3693937	Mb-3	11_6189589	0.7543	-0.0058
Fiber length	T49-20	14_3693937	Mk-8	15_3541748	0.8804	-0.0161
Fiber length	T49-20	14_3693937	Mk-9	15_3543339	0.7543	-0.0058
Fiber length	T49-20	14_3693937	Ml-13	15_521982	0.769	-0.0286
Fiber length	T50-28	14_3739726	Mb-3	11_6189589	0.5348	-0.0032
Fiber length	T50-28	14_3739726	Mk-8	15_3541748	0.4401	-0.0094

Fiber length	T50-28	14_3739726	Mk-9	15_3543339	0.4669	0.0083
Fiber length	T50-28	14_3739726	Ml-13	15_521982	0.5579	-0.0287
Fiber length	T50-28	14_3739726	T49-20	17_3693937	1.0618	-0.0241
Fiber width	Mg-7	12_10871340	Mb-4	11_6189563	0	0
Fiber width	T49-21	14_3700450	Mb-4	11_6189563	0.5143	-0.0194
Fiber width	T49-21	14_3700450	Mg-7	12_10871340	0.6737	-0.0204
Fiber width	T49-22	14_3700644	Mb-4	11_6189563	0.6866	-0.0102
Fiber width	T49-22	14_3700644	Mg-7	12_10871340	0.8459	-0.0346
Fiber width	T49-22	14_3700644	T49-21	14_3700450	1.894	-0.009
Fiber width	T50-30	14_3739748	Mb-4	11_6189563	0.0904	-0.0111
Fiber width	T50-30	14_3739748	Mg-7	12_10871340	0.2984	-0.0378
Fiber width	T50-30	14_3739748	T49-21	14_3700450	0.7833	-0.0305
Fiber width	T50-30	14_3739748	T49-22	14_3700644	1.097	-0.0235
Holocellulos	T49-23	14_3699989	Mb-4	11_6189563	0	0
Holocellulos	T50-28	14_3739726	Mb-4	11_6189563	0.3755	-0.0365
Holocellulos	T50-28	14_3739726	T49-23	14_3699989	0.5348	-0.0609
Holocellulos	T50-31	14_3740162	Mb-4	11_6189563	0.7362	-0.0019
Holocellulos	T50-31	14_3740162	T49-23	14_3699989	0.8955	-0.0264
Holocellulos	T50-31	14_3740162	T50-28	14_3739726	1.1351	-0.0382
Hemicellulose	T50	14_3733576	T49-18	14_3700102	0.4336	-0.0065
Hemicellulose	T50	14_3734278	T49-18	14_3700102	0.3093	0.0089
Hemicellulose	T50	14_3734278	T50-29	14_3733576	0.4656	-0.0153
Hemicellulose	T50-30	14_3739748	T49-18	14_3700102	0.3573	-0.0245
Hemicellulose	T50-30	14_3739748	T50-29	14_3733576	0.4271	-0.031
Hemicellulose	T50-30	14_3739748	T50-32	14_3734278	0.5014	-0.0189
Hemicellulose	T50-33	14_3739833	T49-18	14_3700102	1.1361	0.0007
Hemicellulose	T50-33	14_3739833	T50-29	14_3733576	1.3853	-0.0051
Hemicellulose	T50-33	14_3739833	T50-32	14_3734278	1.1876	-0.0168
Hemicellulose	T50-33	14_3739833	T50-30	14_3739748	1.2923	-0.0075
Hemicellulose	T3-5	1_10706152	T49-18	14_3700102	0.1531	-0.0012
Hemicellulose	T3-5	1_10706152	T50-29	14_3733576	0.4147	0.0005
Hemicellulose	T3-5	1_10706152	T50-32	14_3734278	0.2275	-0.0099
Hemicellulose	T3-5	1_10706152	T50-30	14_3739748	0.4867	-0.0226
Hemicellulose	T3-5	1_10706152	T50-33	14_3739833	1.6545	-0.0058
Lignin	T49-24	14_3695287	Ma-1	4_17508994	0.1531	0.0022
Lignin	T49-25	14_3699030	Ma-1	4_17508994	0.2183	-0.0034
Lignin	T49-25	14_3699030	T49-24	14_3695287	0.5648	-0.0349
Lignin	T49-26	14_3700581	Ma-1	4_17508994	1.095	-0.0071
Lignin	T49-26	14_3700581	T49-24	14_3695287	1.5939	-0.0381
Lignin	T49-26	14_3700581	T49-25	14_3699030	1.7144	-0.0137
Lignin	T50-34	14_3735238	Ma-1	4_17508994	0.3093	0.0089
Lignin	T50-34	14_3735238	T49-24	14_3695287	0.8762	-0.0344
Lignin	T50-34	14_3735238	T49-25	14_3699030	0.2504	-0.0121
Lignin	T50-34	14_3735238	T49-26	14_3700581	1.127	-0.0159

Lignin	T50-35	14_3740094	Ma-1	4_17508994	1.1663	-0.0037
Lignin	T50-35	14_3740094	T49-24	14_3695287	0.9994	-0.0582
Lignin	T50-35	14_3740094	T49-25	14_3699030	1.0819	-0.0332
Lignin	T50-35	14_3740094	T49-26	14_3700581	2.0779	-0.0405
Lignin	T50-35	14_3740094	T50-34	14_3735238	1.1983	-0.0125
Pulp yield	Mk-9	15_3543339	Mb-4	11_6189563	0	0
Pulp yield	Mm-15	12_4950681	Mb-4	11_6189563	0.3638	-0.0236
Pulp yield	Mm-15	13_4950681	Mk-9	15_3543339	0.4552	-0.0358
Pulp yield	T49-27	14_3740532	Mb-4	11_6189563	0	0
Pulp yield	T49-27	14_3740532	Mk-9	15_3543339	0.1107	-0.0161
Pulp yield	T49-27	14_3740532	Mm-15	12_4950681	0.5083	-0.0088

Table S4. Gene-specific primers used in the present study

Primer ID	Forward(5'-3')	Reverse(5'-3')	Purpose
PtoGRAS50-0800	ttgagccgcgccaatatccctATTCGCTGAAATCACCAGTCTCTC	atattggcgcggctcaaTCTCTAGAATTACACGGCGATCTTT	Dual-luciferase reporter assays
PtomiR171a-62SK	gctctagaactagtgTCAGAGAAAACGGGATATTGGTAC	ggcccccctcgaggATGAGTGTACGTGATATTGGCAC	Dual-luciferase reporter assays
PtoGRAS50-pFN19K	cgataacgcgatcgccTGAAGGCCATGCCCCTAC	tgcaggtcgactACACCT CCAAGCAGAAGCTG	DAP-seq
18S-qPCR	GGCATGGAAGGTGATGCAGATC	CTGTGTCAAACAAGAACTTGTCC	RT-qPCR
5.8S-qPCR	ACGTCTGCCTGGGTGTCACGC	GTCTGCCTGGGTGTCACGCAA	RT-qPCR
PtomiR171a- qPCR	TTGAGCCGTGCCAATATCACG	GTCTGCCTGGGTGTCACGCAA	RT-qPCR
PtomiR171k- qPCR	GGATTGAGCCGCGCCAATATC	GTCTGCCTGGGTGTCACGCAA	RT-qPCR
PtomiR171c- qPCR	AGATTGAGCCGCGCCAATATC	GTCTGCCTGGGTGTCACGCAA	RT-qPCR
PtomiR171f- qPCR	CGTGATTGAGCCGTGCCAATATC	GTCTGCCTGGGTGTCACGCAA	RT-qPCR
PtomiR171j- qPCR	CGCCGAGCCGAATCAATATCACT	GTCTGCCTGGGTGTCACGCAA	RT-qPCR
PtoGRAS50- qPCR	GCCCAGAATTAAGCCTTTTCG	AGCAACCAAAACCAACATCAG	RT-qPCR
